# Pemphigus

**Published:** 1907-10-12

**Authors:** 


### Dermatology.

### PEMPHIGUS.

Pemphigus is characterised by the appearance of blebs or bullæ upon the skin, and is a very striking disease. There are not a great many different bullous skin eruptions, so that the diagnosis is fairly certain, provided that one can exclude the following:—

1. Traumatic blebs, such as follow burns and the application of irritants.
2. Pemphigus neonatorum (*a*). This is not a special skin disease at all, but only one of the many manifestations of congenital syphilis.
3. Pemphigus neonatorum (*b*), a contagious bullous eruption of very poor and dirty districts; it is closely related to:—
4. Bullous impetigo.
5. Certain tropical skin diseases.
6. Bullæ, apparently spontaneous, that now and then occur in patients suffering from œdema.

When these six can be excluded, the presence of a bullous eruption constitutes pemphigus, the disease varying considerably in extent and in severity. There may be but a single bulla; more often there are many, coming out in successive crops. Each bulla appears spontaneously without any preceding inflammation—a point in differential diagnosis; it looks exactly like a traumatic blister containing faintly yellow serum which is quite clear unless secondarily infected with staphylococci or other organisms. A day or two after the appearance of the bulla a thin rim of reactionary inflammation appears all round its edge; the raised epidermis of the bleb itself become opaque from absorption of fluid, but should not suppurate; and a raw red surface is exposed if the top of the bleb should become removed. This raw surface is slow to heal, and after healing it leaves behind it a pigmented patch which may remain for months or years. The eruption is as a rule painful, but not itchy. Similar blebs occur in the alimentary canal, particularly in the colon, leading to abdominal pain and diarrhœa. These are the only common symptoms, and in ordinary cases there is no pyrexia. In severe and acute examples of the disease, however, it is not uncommon to find pyrexial attacks, the temperature rising to 104° F., or more; in such instances there is often albuminuria at the same time, and the prognosis is grave. It is true [illegible] may occur even when there is no pyrexia, but the prognosis as regards life is much better when there is no fever than when there is. Pemphigus is often regarded as an extremely dangerous complaint; this is putting the case too strongly, for the majority of the patients do not lose their lives from it; the main trouble is that after one attack a recurrence is very liable to occur in spite of treatment, and repeated recurrences may render the patient's life miserable.

The treatment of pemphigus is essentially by the administration of arsenic internally. An adult should be given 3-minim doses of the liquor arsenicalis four times a day after food to begin with, and provided the drug is well tolerated the dose should be increased every five or six days by 1 minim at a time until a dose of 10 or, in some cases, 15 minims may be reached. The great point to be on the watch for is incipient peripheral neuritis in the legs, any suspicion of which is an indication for stopping the arsenic for the time being. Unfortunately the pemphigus, which is often readily checked and kept under as long as arsenic is being taken, is extremely apt to break out again directly the arsenic is stopped. Local treatment resolves itself into three main parts: (1) Protection from injury; (2) prevention of sepsis; (3) relief of pain. These three essentials are met in most cases by using zinc ointment, to each ounce of which 10 minims of liquefied carbolic acid have been added. It is dangerous to add more carbolic acid than this, because a harmful quantity of it might be absorbed from the raw surfaces.

There is one diagnostic point which may be of great value in differentiating some cases of pemphigus from such things as artefacts, and that is the eosinophilia usually found on examining films of the patient's blood. Eosinophilia in the presence of a bullous eruption is almost conclusive evidence of pemphigus, though the absence of eosinophilia does not exclude it.

The following examples illustrate the four main types that are seen clinically:—

I. *The Recurrent Type* (the most common).—A girl, aged six, came under observation in July for typical pemphigus affecting the trunk, face, and limbs; there was also much pigmentation; no pyrexia and no albuminuria. Liquor arsenicalis was begun in 2-minim doses, gradually increased to 7 minims. Six weeks later she seemed quite well. Arsenic was continued for a time, but was presently stopped, and in October the bullæ recurred.

II. *The Chronic Type.*—A boy, aged six, had never been free from the bullæ since he was a year old. The trouble was better in summer, but exacerbations occurred every winter.

III. *The Acute Type, with recovery.*—A woman, aged 35, who had never suffered from pemphigus before, broke out into bullæ and vesicles almost all over her body and limbs; the only unaffected parts were the scalp, forehead, palms, and soles. There was much pain, but little itching. There was moderate pyrexia and some albuminuria. Diarrhœa was sometimes a trouble, but not constantly. The bullæ began on the wrists, arms, and legs, and for two months fresh blebs kept on appearing. The usual treatment by arsenic was resorted to, the dose increasing to 10 minims; after two months the blebs ceased to reappear, the old ones healed, and up to the present there has been no recurrence.

IV. *The Acute Type, Fatal.*—An elderly lady, was suddenly taken ill with her first atack of pemphigus, the blebs appearing all over the back, shoulders, arms, thighs, and legs. There was no pyrexia and no albuminuria, but within a week the patient was dead. There was an autopsy, and the only internal lesion found was recent pleurisy without pneumonia.

